# Higher diversity and abundance of microRNA-455-5p isoforms confer suppressive role in Wilms’ tumor

**DOI:** 10.3389/fonc.2025.1681367

**Published:** 2025-12-11

**Authors:** Cao Chen, Zhou Zhou, Chunfang Yao, Xiaowei Li, Yifei Wang, Xiaogang Zhou, Hengli Ni

**Affiliations:** 1 Department of Pathology, Children’s Hospital of Soochow University, Soochow University, Suzhou, Jiangsu, China; 2 Department of General Surgery, Children’s Hospital of Soochow University, Soochow University, Suzhou, Jiangsu, China

**Keywords:** Wilms’ tumors, microRNA, isoform, miR-455-5p, *SP100*

## Abstract

**Introduction:**

MicroRNA isoforms (isomiRs) are differentially expressed across tissues, populations, genders, and various disease states, playing significant roles in tumorigenesis and treatment. However, their expression profiles, functions, and clinical relevance in pediatric Wilms' tumour (WT) remain poorly understood.

**Methods:**

Using small RNA-seq data from the TARGET initiative, we profiled isomiRs in WT and adjacent tissues. Bioinformatics tools identified target genes. Key findings were validated in an independent cohort (n=63) using immunohistochemistry (IHC) and multiplex immunofluorescence combined with fluorescence in situ hybridization (mIF-FISH). Associations with histology and survival were analyzed using Kaplan-Meier and Cox regression models.

**Results:**

Our results revealed that various isoforms of miR-455-5p demonstrated greater diversity and abundance in WT compared to its archetype, and were more closely associated with the tumour. Patients exhibiting a higher variety and number of miR-455-5p isoforms showed superior survival rates, independent of tumour differentiation. Notably, the isoform miR-455-5p 0|1 exhibited enhanced complementarity to its predicted target gene *SP100* compared to the archetype, suggesting its potential role in influencing the histological classification of WT through *SP100* regulation.

**Discussion:**

This study provides the first comprehensive analysis of isomiRs in WT, identifying miR-455-5p isoform diversity as a novel prognostic indicator. The specific isoform miR-455-5p 0|1 likely exerts a tumour-suppressive role, potentially through a non-canonical regulatory interaction with *SP100*. These findings unveil isomiR-mediated regulation as a critical layer of molecular complexity in Wilms’ tumour, offering new insights for prognostic stratification.

## 1 Introduction

MicroRNAs (miRNAs) are small non-coding RNAs that play crucial regulatory roles in various biological processes, including tumorigenesis (1, 2). During their study of miRNA expression in human embryonic stem cells using deep sequencing in 2008, Morin et al. introduced the term ‘isomiRs’ for miRNAs that differ from their archetype sequence (3). IsomiRs, referring to miRNA isoforms with sequence variations at their 5’ or 3’ ends, are produced mainly due to imprecise cleavage by Drosha or Dicer during miRNA biogenesis. The inception of miRNA profiling revealed sequence variants at the 5’ and 3’ ends, initially dismissed as artifacts and excluded from downstream analysis (4). However, this phenomenon was soon recognized as an integral component of the miRNA biogenesis landscape (5). These variants were consequently termed isomiRs and have become a primary focus of miRNA analytical tools (6). As sequencing data continues to accumulate, mounting evidence underscores the significance of these isoforms across various human diseases, including cardiovascular, neurodegenerative, and psychiatric disorders, as well as chronic inflammatory diseases (7, 8). Notably, analyses of isomiR expression patterns demonstrate the ability to distinguish tumor cells from their normal counterparts and to further differentiate between diverse tumor types and subtypes (9, 10). This compellingly suggests that isomiRs hold substantial potential as valuable diagnostic and prognostic biomarkers in oncology (11, 12), thereby establishing them as crucial and reliable molecules detectable in modern small RNA-seq data.

Wilms’ tumor, also known as nephroblastoma, constitutes 5% of pediatric malignancies and accounts for 95% of malignant kidney tumors diagnosed in childhood, representing a cancerous growth affecting the kidney (13). The standard approaches for treating WT encompass surgery, radiotherapy and chemotherapy (14). However, the development of metastasis or recurrence presents a major clinical challenge and is associated with a dismal prognosis (15). Increasing evidence suggests that miRNAs play a crucial role in the initiation and progression of WT (16). Through whole-genome miRNA-seq profiling analysis, dysregulation of miRNA expression has been identified in WT (17).

For this project, we conducted a comprehensive analysis of miRNA isoforms in WT using data from TARGET. Following this analysis, SP100, which has been implicated in transcriptional regulation and interferon responses (18), was identified as having a strong association with miR-455-5p 0|1, marking it as a primary target for our subsequent *in silico* and *in vitro* investigations. Therefore, this study aims to characterize the abundance and expression profiles of various isomiRs across Wilms tumor histological subtypes and to provide insights for novel therapeutic strategies.

## 2 Methods

### 2.1 Bioinformatic analysis

The small RNA-seq raw read counts and corresponding clinical data for Wilms’ tumor patients were downloaded from the GDC-TARGET database (https://portal.gdc.cancer.gov/projects/TARGET). This dataset comprises 127 tumor and 5 adjacent non-tumorous tissue samples. To indicate each isomiR, we combined its common name (e.g., miR-455-5p), and two numbers separated by a vertical bar, for example, miR-455-5p 0|1. The first number indicates the relative position of the isomiR’s 5’ terminus with respect to the archetype’s 5’ end, and the second number indicates the analogous relationship for the isomiR’s and the archetype’s 3’ termini. The positive (+) or negative sign (−) indicate the isomiR’s terminus is downstream or upstream from the archetype terminus, respectively. So, miR-455-5p 0|0 denotes the archetype miRNA that arises from the 5p arm of the miR-455 precursor. For quality control, we considered only isomiR that had more than 100 reads in at least one sample, the methodology is as before (19).

Target genes of miR-455-5p were identified using the R package multiR and the miRanda database. To ensure high-confidence predictions, only genes that were either experimentally validated or concurrently predicted by both tools were selected for subsequent analysis. The 3’ UTR sequences of these 258 target genes were then extracted and analyzed with RNA22 to identify miRNA response elements and to compare the binding difference between miR-455-5p 0|0 and miR-455-5p 0|1. One nt mismatch in the seed region was allowed. Interactions with a p-value < 0.05 were defined as effective (**Supplementary Tables 1**, **2**).

### 2.2 Immunohistochemistry

IHC was performed to detect the presence and distribution of SP100 protein in Wilms’ tumor and adjacent non-tumorous tissues. Formalin-fixed, paraffin-embedded tissues were sectioned at 3-μm thickness. Following deparaffinization, rehydration, and antigen retrieval, the sections were blocked with 5% BSA. Subsequently, they were incubated overnight at 4 °C with a rabbit anti-SP100 primary antibody (Proteintech, 11377-1-AP, 1:100) at 1:100 dilution. After washing, the sections were treated with an HRP-conjugated goat anti-rabbit secondary antibody (ZSGB, PV-6001) for 30 minutes at 37 °C.Following DAB visualization, the sections were counterstained with hematoxylin, dehydrated, cleared, and mounted.

### 2.3 Multiplex immunohistochemistry and fluorescence *in situ* hybridization

We performed mIHC for SP100, followed by FISH for miR-455-5p 0|1. FISH was performed using FISH kits and a Cy3 labeled miR-455-5p 0|1 antisense probe (Sequence: TATGTGCCTTTGGACTACATCGT, GenePharma, China). Briefly, slides were baked at 65°C for 30 minutes, deparaffinized, and boiled in purified water. SP100 antibody (Proteintech, 11377-1-AP, 1:100) was applied overnight at 4°C, followed by goat anti-rabbit antibody (Absin, China) at 37°C for 30 minutes. After washing, amplifier solution containing fluorochrome (Absin, China) was added and incubated at 37°C for 30 minutes. Slides were treated with pepsin, dehydrated in ethanol, and hybridized with the probe at 37°C for 18 hours. Then, they were washed in 2×SSC and 0.1% NP-40/2×SSC, stained with DAPI, and imaged under a fluorescence microscope (Nikon Ni-U, Japan).

### 2.4 Tumor tissue and clinical information

A total of 63 surgical samples from patients with Wilms’ tumor were collected at the Department of Pathology at Children’s Hospital of Soochow University from April 2015 to April 2025. The diagnosis of Wilms’ tumor and the critical histological classification into favorable or unfavorable subtypes were rigorously confirmed by two independent senior pathologists according to the current standard guidelines. The samples included favorable (n = 22), unfavorable (n = 33), and normal control (n = 8) cases. The study was conducted with the informed consent and signed by the family members of the children, and the specimen collection was considered and approved by the Medical Ethics Committee of the hospital.

### 2.5 Evaluation of IHC staining

The stained sections were evaluated independently by two pathologists who were blinded to the clinical data, with immunoreactivity quantified using both the Immunoreactivity Score (IRS, intensity × proportion) and the H-score. The staining intensity was scored as 0 (negative), 1 (weak), 2 (moderate), or 3 (strong). The proportion of positive tumor cells was scored as 0 (0%), 1 (1-25%), 2 (26-50%), 3 (51-75%), or 4 (76-100%). The following primary antibodies were used: anti-SP100 (11377-1-AP, Proteintech, USA).

### 2.6 Quality control and isomiR expression analysis

To ensure data robustness, low-abundance isomiRs were filtered out by retaining only those with >100 raw reads in at least one sample. Given an average sequencing depth of 8.9 million reads per sample, this threshold corresponds to approximately 12 reads per million (RPM).

IsomiR diversity was analyzed by focusing on miRNAs with a median number of isoforms ≥ 3 across all samples. For each miRNA, samples were categorized into “more” or “less” groups based on whether their isoform count was above or below the median, respectively. Based on our initial miRNA-seq profiling, we selected candidates for further investigation by applying a threshold of Fold Change (T/N) > 2. This analysis identified hsa-miR-455-5p and hsa-miR-582-5p as the top candidates meeting this threshold. Given the clinical relevance of hsa-miR-455-5p in subsequent histological classification, it was selected for further investigation.

### 2.7 Statistics

For continuous variables, differences between two groups were assessed using the Student’s t-test, while differences among more than two groups were evaluated by one-way ANOVA. For categorical variables, the Chi-square test was applied to find the difference between different groups. Survival analysis was estimated by the Kaplan-Meier method which provides a non-parametric estimate of the survival function over time, and the differences between curves were compared with the log-rank test. Univariate Cox regression was performed to estimate HR (hazard ratio). To adjust for potential confounding and identify independent prognostic factors, the tumor histology classification was added as the covariant in the multi-variant Cox regression model. All statistical analyses were performed using R software (version 4.4.1). A two-sided p-value < 0.05 was considered statistically significant.

## 3 Results

### 3.1 More microRNAs isoforms’ variety in Wilms’ tumors

**Figure 1:**
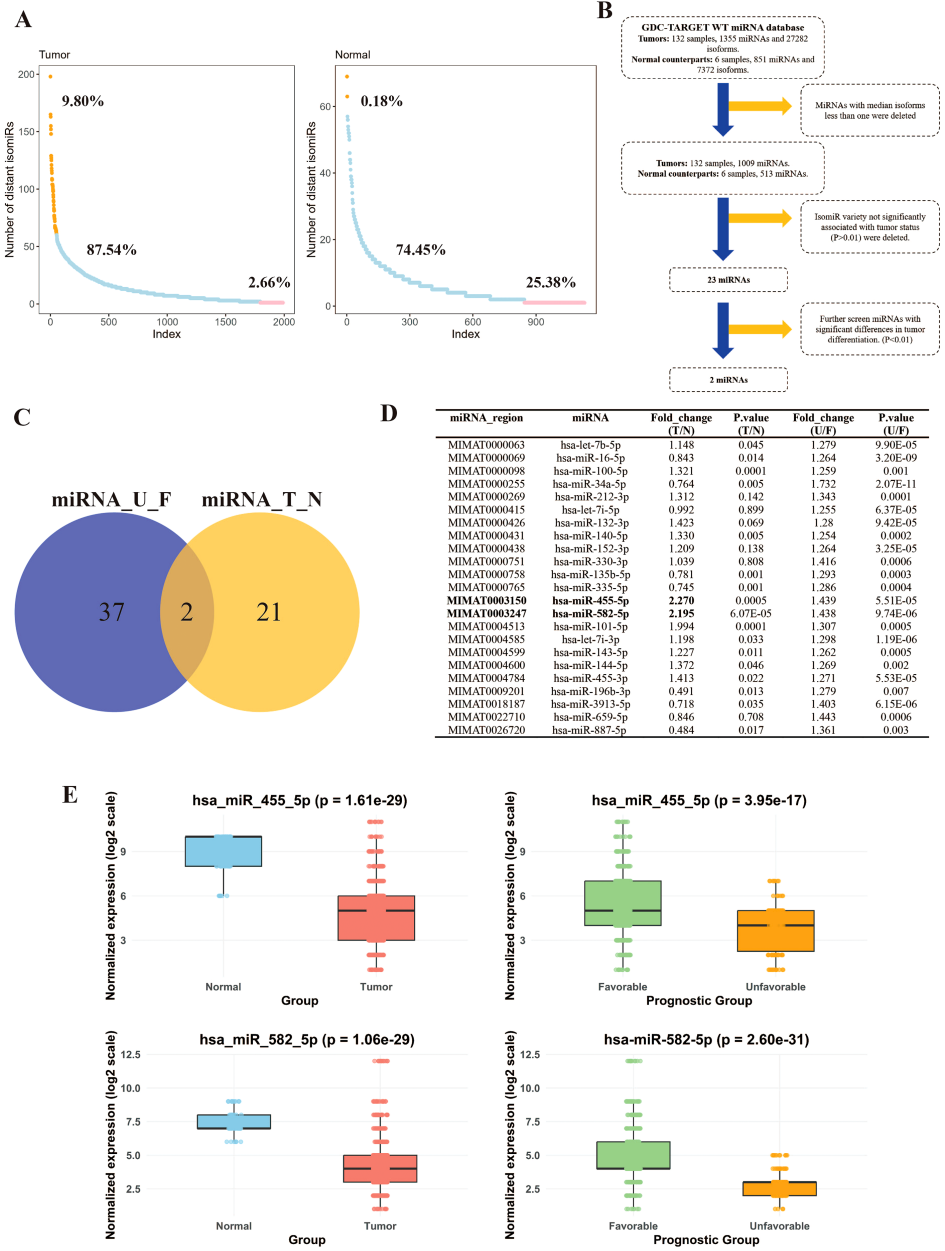
MiRNA isoform variety correlates with the malignancy of Wilms’ tumor. **(A)**. The number of distinct isomiRs per tissue derived from TARGET in tumor tissues (up) and the normal counterparts (down). (Orange: miRNAs with isoforms per tissue more than 10; Blue: miRNAs with isoforms per tissue between 2 to 10; Pink: miRNAs with isoforms per tissue less than 1) according to data from TARGET; **(B)**. Filtering process to identify WT malignancy associated miRNAs by comparing the variety of isoforms between tumor and normal tissues or between unfavorable and favorable tumor samples; **(C)**. The Venn diagram displays the intersection of differentially expressed genes between two parts. **(D)**. The 23 iso-miRNAs altered significantly different (*P* < 0.01) in tumor tissues compared with normal counterparts. Among them, the isoform number of miR-455-5p and miR-582-5p change significantly more than 2-fold. **(E)**. The comparison of the types of isomers in hsa_miR_455 and hsa_miR_582 based on two different grouping scenarios, namely Normal/Tumor and Favorable/Unfavorable.

Based on GDC-TARGET data, we counted the isoform number of each miRNA in tumor and para-tumor tissue (**Figure 1A**). In tumor tissues, 9.80% (195/1990) miRNA showed only one isoform, 87.54% (1742/1990) between 2 and 9 isoforms, and 2.66% (53/1990) miRNA harbored more than 10 isoforms. In the non-tumorous tissues’ counterparts, 25.38% (287/1131) miRNA with one single isoform, 74.45% (842/1131) between 2 and 9 isoforms, and 0.18% (2/1131) miRNA presented more than 10 isoforms. There is significant difference of the general isoform variety between tumor and non-tumorous tissues (*P* < 0.001).

To identify certain miRNAs with isoforms variety associated with tumor malignancy, we filtered the data as shown in **Figure 1B**. We first identified 23 miRNAs with significant differences in isoform number between tumor and normal tissues (miRNA_T_N comparison). Subsequently, we compared the diversity of isomiRs within tumors between those with favorable and unfavorable (miRNA_U_F) and found that 39 miRNAs showed significant differences between the two groups (**Figures 1 C, D**). Of these, only 2 miRNAs, miR-455-5p and miR-582-5p, were commonly shared. Both had less variants in Wilms’ tumor tissues or in unfavorable tumors than in normal tissue or in favorable tumors (**Figure 1E**).

### 3.2 Patients with higher miR-455-5p isoforms’ variety exhibited favorable survival

**Figure 2:**
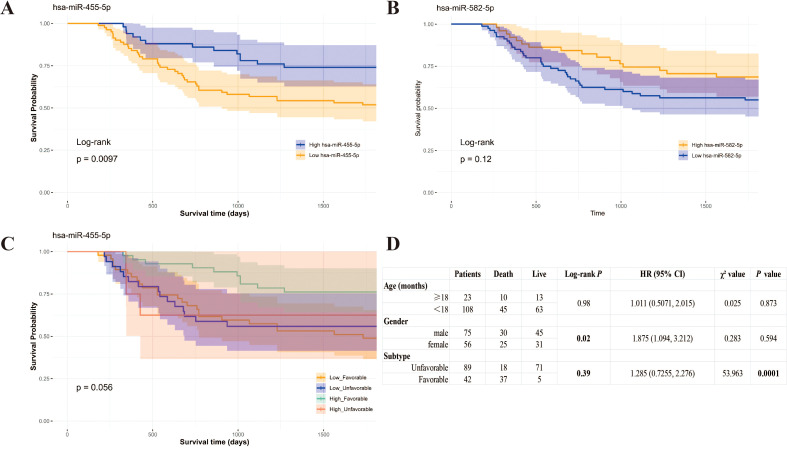
More variety of miR-455-5p is favorable for WT patients’ survival. **(A, B)**. Kaplan-Meier plot of WT survival about the isoform variety of miR-455-5p **(A)** and miR-582-5p **(B, C)** Kaplan-Meier plot of WT survival by different quantity groups of miR-455-5p combining tumor histology classification; **(D)** Univariate survival analysis of clinical factors from TARGET data.

Survival analysis revealed that Wilms’ tumor patients with higher diversity of miR-455-5p isoforms experienced significantly better 5-year event-free survival (*P* = 0.0097; **Figure 2A**). In contrast, the diversity of miR-582-5p isoforms showed no significant association with patient prognosis (*P* = 0.12; **Figure 2B**).

Using the histological classification (favorable vs. unfavorable) as a covariate, we found that miR-455-5p isoform diversity predicted survival in Wilms tumor patients. Those with higher isoform diversity and a favorable prognosis had a better clinical outcome (**Figure 2C**). Survival analysis revealed that subtype and gender were significant factors in predicting patient survival (**Figure 2D**). Therefore, the variety of miR-455-5p isoforms was higher in adjacent non-tumorous tissues, as well as in patients with favorable survival outcomes.

### 3.3 The quantity of miR-455-5p isomiRs correlated with the malignancy of Wilms tumors

**Figure 3:**
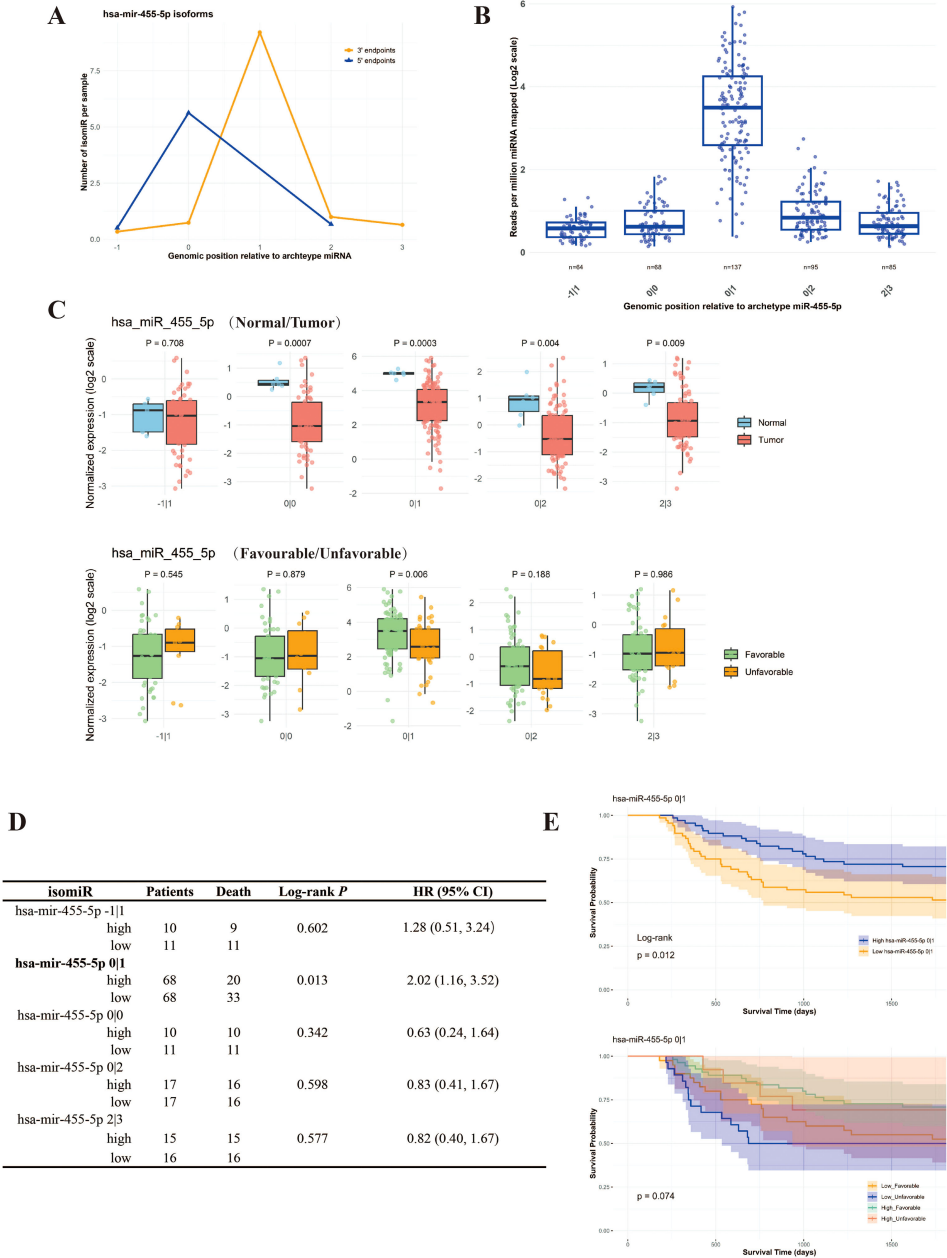
Lower quantity of miR-455-5p isoforms correlates with WT malignancy. **(A)** The distribution of the isoform number of miR-455-5p endpoints at each genomic position relative to the archetype’s coordinates; **(B)** Relative expression of different miR-455-5p isoforms; **(C)** The expression of the five isoforms of miR-455-5p in different tissue groups (tumor and its normal counterparts) (up) and in different tumor histology classification (unfavorable and favorable) (down); **(D)** Univariate Cox regression of different iso-miR-139-5p in WT’s survival; **(E)** Kaplan-Meier plot of WT survival by different quantity groups of miR-455-5p 0|1 (up) or combining tumor histology classification (down).

**Figure 3A** shows the distribution of the 5’ and 3’ termini of the isomiRs around the termini of the archetype miR-455-5p. As expected, the 3’ ends were less conserved than the 5’ ends. **Figure 3B** shows the expression level of each specific miR-455-5p isoform, revealing that isoform 0|1 exhibited the highest expression level among the five isoforms. We then compared the expression levels of iso-miR-455-5p in cancerous and adjacent non-tumorous tissues (**Figure 3C** up) and WT tissues different histology (favorable vs. unfavorable) (**Figure 3C** down). Each miR-455-5p isoform showed lower expression in adjacent non-tumorous tissues, except for miR-455-5p -1|1.

Furthermore, only miR-455-5p 0|1 was found to be correlated with the histological classification in WT tissues (**Figure 3C**). Of these, the highest isoform, miR-455-5p 0|1, was found to significantly correlate with patient survival when tumor histology classification was taken into account (**Figure 3D**). Survival analysis also showed that a higher level of miR-455-5p 0|1 was favorable for WT survival (**Figure 3E**).

### 3.4 Different miR-455-5p isoforms showed different regulatory effects on its target gene *SP100*

**Figure 4:**
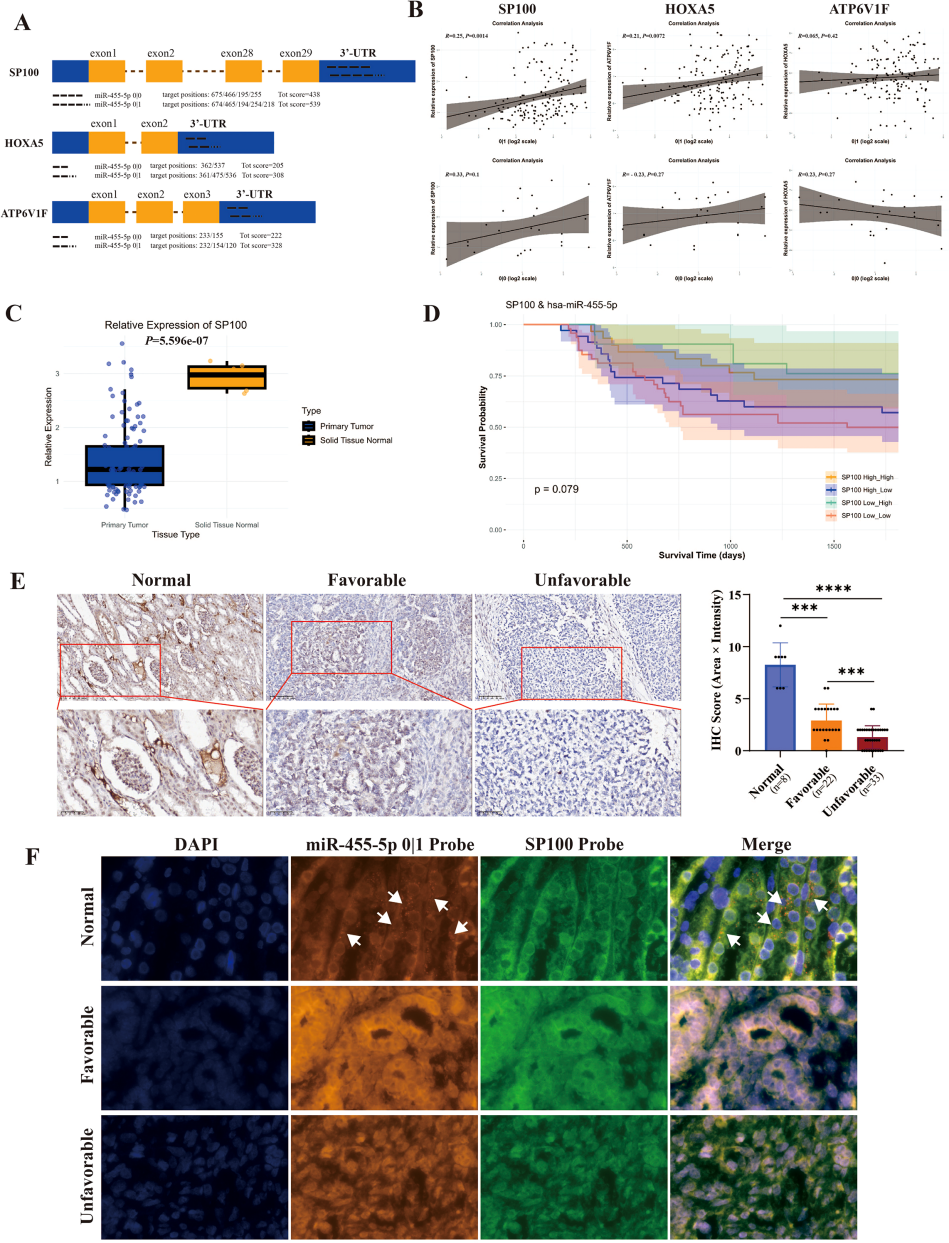
MiR-455-5p 0|1 and the archetype show different regulatory effect on SP100**(A)** Potential binding site of miR-455-5p 0|0 and 0|1 in the 3’untranslated region (UTR) of *SP100*, *HOXA5* and *ATP6V1F*. **(B)** The Pearson correlation analysis of miR-455-5p 0|0 (or 0|1) and *SP100*, *HOXA5*, *ATP6V1F*. **(C)** The relative expression of *SP100* in primary tumor and solid tissue normal. **(D)** Kaplan-Meier plot of WT survival by different quantity groups of SP100 combining miR-455-5p 0|1. **(E)** The expression levels of SP100 in normal tissues (n = 8), WT tumors favorable (n = 22) and unfavorable (n = 33). **(F)** The co-localization of SP100 and miR-455-5p 0|1 was verified through mIF experiments.

A systematic search was conducted for target genes of the isomiRs of miR-455-5p, with particular interest arising in *SP100*, *HOXA5* and *ATP6V1F*. Furthermore, the analysis revealed that *HOXA5* and *ATP6V1F* possess a reduced number of binding sites for miR-455-5p 0|1 in comparison to *SP100*. In the predicted target combination of MiR-455-5p and SP100, MiR-455-5p 0|1 shows 5 target sites, while the prototype miR-455-5p contains 4 target sites (**Figure 4A**). Furthermore, an investigation was conducted into the correlation between SP100 and miR-455-5p 0|1, utilizing data from the TARGET database. This analysis yielded a correlation curve, from which it was determined that the most significant correlation was observed between SP100 and miR-455-5p 0|1 (**Figure 4B**). Consequently, the present study concentrated on examining the interaction between miR-455-5p 0|1 and SP100.

The results of this study demonstrate that SP100 is significantly low-expressed in tumor tissues (**Figure 4C**). A joint analysis of the expression levels of SP100 and miR-455-5p 0|1 was conducted, and survival curves were plotted. The results indicated that patients with low expression of both SP100 and miR-455-5p 0|1 had decreased survival rates compared to other groups (**Figure 4D**).

IHC showed that SP100 expression was high in normal tissues but significantly reduced in tumors, especially those with poor prognosis. A significant difference in SP100 expression was observed among normal, favorable, and unfavorable tumor tissues (**Figure 4E**). Utilizing the mIF technique, a comparison was made between the target effect of iso-miR-455-5p and SP100. The results indicated that miR-455-5p 0|1 is distributed in proximity to SP100 (**Figure 4F**), thereby suggesting a potential positive interaction between them.

## 4 Discussion

MicroRNAs are pivotal post-transcriptional regulators implicated in Wilms’ tumor (WT) pathogenesis (20, 21), with well-characterized roles for species such as miR-21 and miR-155 (22–24). However, the landscape and functional significance of isomiRs in WT have remained entirely unexplored. Our study addresses this gap by systematically profiling isomiRs in WT, leading to the identification of miR-455-5p and its specific isoform, miR-455-5p 0|1, as key players with clinical relevance.

Although bioinformatics tools exist to identify isomiRs (25), the functional impact of this heterogeneity remains unclear. The miRNA regulatory network is inherently complex, with multi-targeting and cooperativity (26), and miRNA function can vary by context, as seen with miR-455-5p in different cancers (27). Here, we extend this paradigm by demonstrating that even different isoforms of the same miRNA, specifically the miR-455-5p archetype and its 0|1 isomiR, can exert distinct regulatory effects on a common target, *SP100*. This finding highlights how isomiR generation can functionally diversify the miRNA regulatory network.

The target of this differential regulation, SP100 (Speckled Protein 100), is a chromatin-associated “reader” that plays pivotal roles in interpreting the epigenome and enabling cell-specific transcriptional programs (28). Its intimate connection with oncogenic processes is underscored by studies showing that SP100 expression can reduce the malignancy of glioma tumors (29). Furthermore, SP100 has been reported to inhibit the HPV life cycle (30). Therefore, the precise regulation of *SP100* by specific miR-455-5p isoforms, as uncovered in our study, may represent a novel layer of control in cellular physiology and disease pathogenesis.

Our key finding is the significant positive correlation between the miR-455-5p 0|1 isoform and elevated expression of the tumor suppressor SP100. This positive regulatory relationship challenges the canonical model of miRNA-mediated repression. *In bioinformatics* analysis suggests that the 0|1 isoform may possess a stronger binding affinity for the enhancer region of SP100, potentially representing a novel, non-canonical mechanism for gene activation. This unexpected functional diversification through isoform generation significantly expands the complexity of the miRNA regulatory network and presents a compelling mechanism for future investigation.

In conclusion, our study transcends the conventional miRNA paradigm by revealing the functional significance of a specific isomiR, miR-455-5p 0|1, in Wilms’ tumor. We establish a novel, positive regulatory relationship between this isoform and the tumor-suppressive SP100 protein, which is associated with favorable patient outcomes. While the precise mechanism invites further exploration, our work unequivocally positions isomiR biology as a crucial and previously overlooked layer of regulatory complexity in pediatric renal cancer, offering new potential avenues for prognostic stratification.

## Data Availability

The original contributions presented in the study are included in the article/**Supplementary Material**. Further inquiries can be directed to the corresponding authors.
